# DNA damage-induced activation of CUL4B targets HUWE1 for proteasomal degradation

**DOI:** 10.1093/nar/gkv325

**Published:** 2015-04-16

**Authors:** Juan Yi, Guang Lu, Li Li, Xiaozhen Wang, Li Cao, Ming Lin, Sha Zhang, Genze Shao

**Affiliations:** 1 Department of Cell Biology, School of Basic Medical Sciences, Peking University, Beijing 100191, China; 2 Institute of Systems Biology, Peking University, Beijing 100191, China; 3 Department of Breast Surgery, the First Hospital of Jilin University, Changchun 130021, China

## Abstract

The E3 ubiquitin ligase HUWE1/Mule/ARF-BP1 plays an important role in integrating/coordinating diverse cellular processes such as DNA damage repair and apoptosis. A previous study has shown that HUWE1 is required for the early step of DNA damage-induced apoptosis, by targeting MCL-1 for proteasomal degradation. However, HUWE1 is subsequently inactivated, promoting cell survival and the subsequent DNA damage repair process. The mechanism underlying its regulation during this process remains largely undefined. Here, we show that the Cullin4B-RING E3 ligase (CRL4B) is required for proteasomal degradation of HUWE1 in response to DNA damage. CUL4B is activated in a NEDD8-dependent manner, and ubiquitinates HUWE1 *in vitro* and *in vivo*. The depletion of *CUL4B* stabilizes HUWE1, which in turn accelerates the degradation of MCL-1, leading to increased induction of apoptosis. Accordingly, cells deficient in CUL4B showed increased sensitivity to DNA damage reagents. More importantly, upon *CUL4B* depletion, these phenotypes can be rescued through simultaneous depletion of *HUWE1*, consistent with the role of CUL4B in regulating HUWE1. Collectively, these results identify CRL4B as an essential E3 ligase in targeting the proteasomal degradation of HUWE1 in response to DNA damage, and provide a potential strategy for cancer therapy by targeting HUWE1 and the CUL4B E3 ligase.

## INTRODUCTION

The human genome is frequently challenged by many types of DNA damage resulting from both exogenous (environmental factors such as ultraviolet [UV]/ionizing radiation [IR]) and endogenous (cellular metabolic processes) sources (1). To counteract these threats, cells have evolved an intrinsic mechanism—the DNA damage response (DDR)—to ensure genome integrity (2). In response to DNA damage, the cell cycle checkpoint is activated, leading to cell cycle arrest. Meanwhile, DNA damage repair pathways are initiated. In addition, DNA damage also induces apoptosis to remove any cells with unrepaired DNA lesions. These processes are interwoven and highly coordinated to maintain genome integrity.

During the DDR process, many key proteins involved are rapidly degraded or turned over through the ubiquitin-proteasome pathway. Numerous E3 ligases have been reported to play a critical role in regulating DDR, possibly through the targeting of essential factors that control these processes, leading to ubiquitination (2–6). The canonical protein ubiquitination process requires the participation of a series of enzymes, including E1 (ubiquitin-activating enzyme), E2 (ubiquitin-conjugating enzyme) and E3 (ubiquitin ligase) (7). E3 ligases are crucial in determining the substrate specificity during protein ubiqutination, thereby playing significant roles in the modulation of DDR.

HUWE1 (also known as Mule, ARF-BP1, E3Histone, UREB1, HECTH9 and LASU1) is a HECT (homology to E6-AP C terminus) E3 ubiquitin ligase that is involved in diverse cellular processes, including apoptosis (8), DNA replication, DNA damage repair (9–13) and transcriptional regulation (14–18). Among those reported functions, apoptosis and DNA damage repair seem to be the major functions that are mediated by HUWE1. Increasing evidence shows that HUWE1 is a pivotal regulator of DNA damage-induced apoptosis. The most important substrate of HUWE1 identified so far is MCL-1, a BCL-2 family member that is essential to the inhibition of apoptosis. It has been demonstrated that HUWE1-mediated ubiquitination and proteasomal degradation of MCL-1 are required to induce apoptosis in response to DNA damage (8). Another HUWE1 substrate involved in apoptosis is mitofusin 2 (Mfn2), an essential component of the fusion apparatus of the mitochondrial outer membrane. Under conditions of stress, HUWE1 targets Mfn2 for ubiquitination and proteasomal degradation, resulting in mitochondrial fragmentation, thereby leading to enhanced apoptotic cell death (19). Another apoptosis-related protein, RASSF1C, was also identified as a HUWE1 substrate (20). In addition, p53, a key effector in stress-induced apoptosis, was found to be regulated by HUWE1 through the ubiquitin-proteasomal system (UPS) (18). Previous studies have shown that HUWE1 is essential for DNA damage-induced p53 activation and apoptosis induced by histone deacetylase (HDAC) inhibitors (21). Recently, we discovered that HUWE1 mediates BRCA1 ubiquitination and degradation (13). Collectively, these findings highlight a critical role of HUWE1 in the mediation of stress-induced apoptosis and DNA damage repair.

Given the critical role of HUWE1 in stress-induced apoptosis and DNA damage repair, it is of great significance to understand how its activity and protein turnover is regulated during the DDR process. Previous studies have shown that HUWE1 is downregulated in response to genotoxic stress (17,22). It has been shown that the E3 ligase activity of HUWE1 can be inhibited by Alternative reading frame (ARF) (18), a tumor suppressor that can be activated through hyperproliferative signals (23). However, a study from Kamijo has confirmed that ARF-null cells are still capable of elevating p53 levels in response to IR, suggesting the existence of an ARF-independent pathway for the downregulation of HUWE1, in response to DNA damage (24). Recent studies have shown that HUWE1 downregulation following DNA damage is dependent on its self-ubiquitination and on subsequent proteasomal degradation, and is promoted by the inactivation of the deubiquitination enzymes USP7S and USP4 (17,18,22). Despite these findings, it is unclear whether other pathways, especially those involving E3 ligases, are involved in regulating HUWE1.

The Cullin4-RING ubiquitin ligases (CRL4) are representatives of a superfamily of E3 complexes that are assembled with Cullin4, DDB1 and ROC1 (25), and are involved in a wide variety of cellular processes, including cell cycle control, DNA replication and DDR (26). In humans, there are two Cullin 4 members, CUL4A and CUL4B. They share extensive sequence homology, playing essential roles in the maintenance of cell growth and in the targeting of certain CUL4 substrates for ubiquitination (27). CUL4B contains a unique nuclear localization signal at its N terminus, which is absent in CUL4A and other Cullins (28,29), and displays a distinct function from CUL4A. Specifically, CUL4B is responsible for the ubiquitination and the degradation of sex steroid receptors (30). A recent study showed that a mutation in *CUL4B* results in X-linked mental retardation (31,32).

Similar to other Cullins, CRL4B activity is regulated through neddylation (33). Neddylation is a process whereby the NEDD8 protein is conjugated to its target proteins, which is analogous to ubiquitination and which relies on the specificity of the E1, E2 and E3 enzymes (34,35). Until recently, only Rbx1, Tfb3 and members of the DCN1 families had been identified as NEDD8 E3 ligases (36–40). DCNL3, a member of the DCN1 family, has been showed to be upregulated in cancer cell lines that had been treated with UVC irradiation, suggesting that the neddylation-induced activation of CRLs may be of significance in modulating the DDR process through the targeting of essential DDR regulators (41). However, the identity of these substrates remains undefined.

In this study, we show, for the first time, that CRL4B regulates HUWE1 stability through the ubiquitin-proteasome pathway in response to DNA damage. CUL4B is activated through neddylation in response to DNA damage, and targets HUWE1 for ubiquitination and subsequent proteasomal degradation. This study sheds light on the mechanism through which HUWE1 is regulated in response to DNA damage, and has important implications for cancer therapy.

## MATERIALS AND METHODS

### Plasmids

Plasmids *pcDNA3.1-Flag-HA-HUWE1* and *pEYFP-HUWE1*, *pcDNA3.1-Flag-HA-CUL4B* were generated by a polymerase chain reaction-based subcloning strategy using *pFast-Bac-Mule* or *pcDNA3-Myc-CUL4B* plasmids as templates. *pcDNA3-ARF-BP1-V5-His* was provided by Wei Gu (Columbia University), and *pFast-Bac-Mule* was provided by Xiaodong Wang (UT Southwestern Medical Center). *pcDNA3-Myc-CUL4B* was kindly provided by Dr Yue Xiong, University of North Carolina at Chapel Hill, and Dr Qunying Lei, Fudan University. *pcDNA3-Myc-CUL4B K859R* mutant plasmid was generated by site-directed mutagenesis and verified by DNA sequencing. *pFastBac1-GST-Huwe1-1-2500* was generated by introducing a Glutathione S-transferase (GST)-tag fused *HUWE1* DNA fragment encoding amino acids 1–2500 of HUWE1 protein. Additional details are provided in Supplementary Materials and Methods. Primer sequences of subcloning and mutation are shown in Supplementary Table S1.

### Cells, cell culture and DNA damage treatment

Human embryonic kidney HEK-293T, HeLa, HeLaS3, MCF-7 and U2OS cells were maintained in DMEM (Gibco) supplemented with 10% FBS (Hyclone), 100 mg/ml penicillin and 100 mU/ml streptomycin in 5% CO_2_ at 37°C. All the above cell lines were obtained from the American Type Culture Collection. Cells were treated with different DNA damage regents (doxorubicin, etoposide or 8-Gy IR) and harvested at the indicated time. Additional details are provided in Supplementary Materials and Methods.

### Transfection and stable cell lines

Lipofectamine RNAi MAX and Lipofectamine 2000 reagent (Invitrogen) were used for transient knockdown by siRNA or transient overexpression, respectively. Target sequences of siRNAs are shown in Supplementary Table S2. A detailed description of the experimental procedures and the generation of FH-CUL4B S3 HeLa stable cell lines are available in the Supplemental Materials and Methods.

### Immunoprecipitation and immunoblotting

A detailed description of the experimental procedures is available in the Supplemental Materials and Methods. The expression levels of proteins in immunoblotting were quantified using Image J and normalized against that of tubulin from at least three independent experiments. Detailed information about protein quantification using Image J is available in the Supplemental Materials and Methods.

### Cycloheximide chase experiment

293T and HeLa cells were transfected with plasmids or siRNAs, treated with cycloheximide (100 mg/ml) and then subjected to immunoblot analysis. A detailed description is available in the Supplemental Materials and Methods.

### *In vivo* and *in vitro* ubiquitination assays

*In vivo* and *in vitro* ubiquitination assay were performed as described in Supplemental Materials and Methods.

### Cell viability and apoptosis assays

Cell viability was assessed indirectly by MTT assay. Apoptosis assay was detected by Annexin V/PI double staining. A detailed description of the experimental procedures is available in the Supplemental Materials and Methods.

## RESULTS

### The downregulation of HUWE1 in response to DNA damage is accompanied by the activation of CUL4B

**Figure 1.**
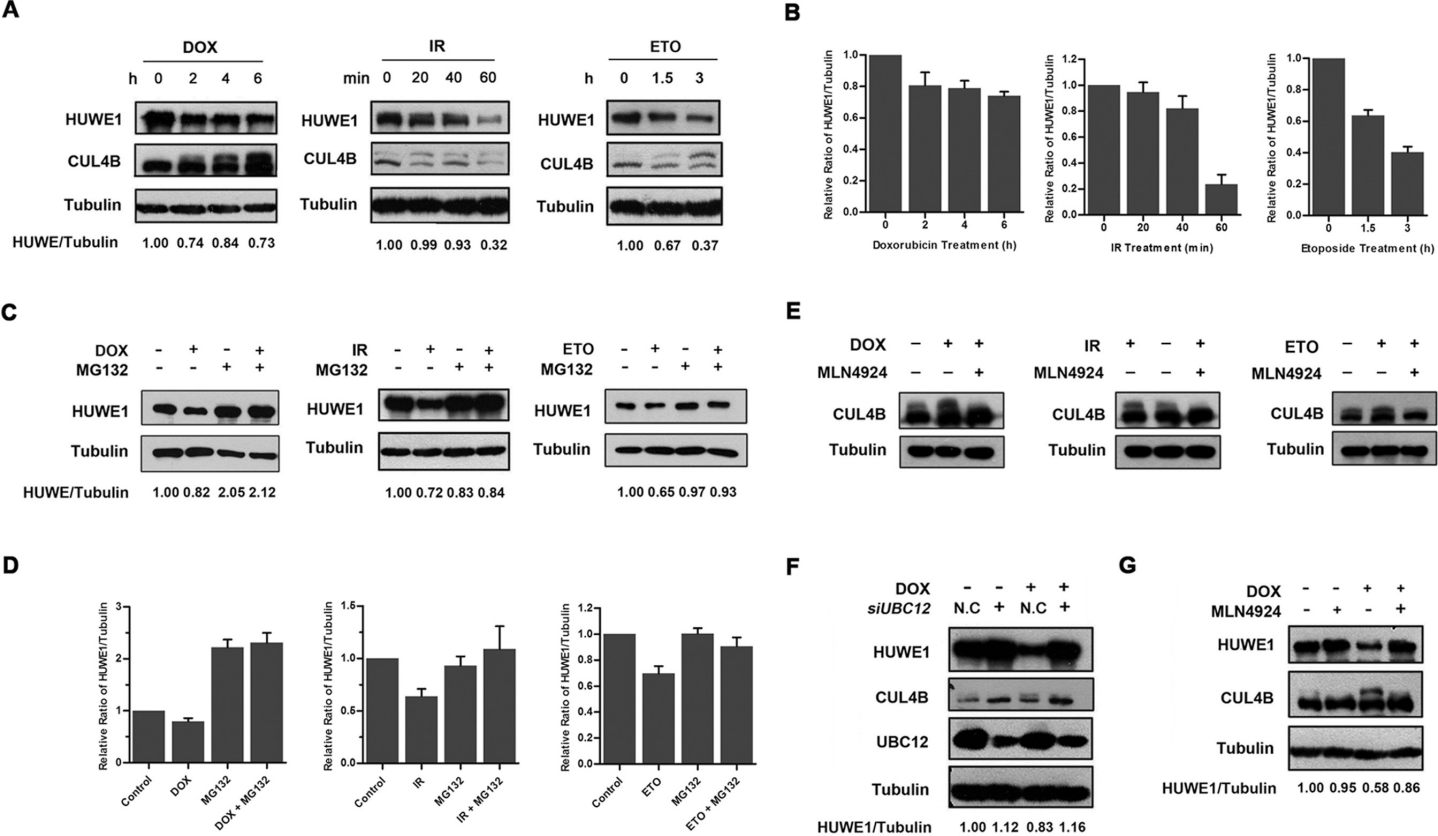
Activation of CUL4B is associated with an increased proteasomal degradation of HUWE1 in response to DNA damage. (**A**) HeLa cells were treated with doxorubicin (0.5 μg /ml), IR (8-Gy) or etoposide (10 μM) and harvested at the indicated time points post treatment. (**B**) shows quantification of A. Columns, mean; Bars, ± S.D. (**C**) HeLa cells were exposed to doxorubicin (0.5 μg /ml), IR (8-Gy) or etoposide (10 μM) and treated with 20 μM proteasome inhibitor MG-132 for 4 h. (**D**) shows quantification of C. Columns, mean; Bars, ± S.D. (**E**) HeLa cells were exposed to doxorubicin (0.5 μg /ml), IR (8-Gy) or etoposide (10 μM) and treated either with or without MLN4924 (1.5 μM) for 4 h. (**F**) HeLa cells were transfected with *UBC12* siRNA for 48 h as descried in Experimental Procedures, and were then treated with or without doxorubicin (0.5 μg/ml) 4 h before being harvested. (**G**) HeLa cells were harvested 4 h post treatment with 0.5 μg /ml doxorubicin, 1.5 μM MLN4924, and 0.5 μg /ml doxorubicin plus 1.5 μM MLN4924, respectively. All the samples in A, C, E–G were analyzed by western blotting with specific antibodies for the indicated proteins.

To examine the effect of DNA damage on the protein levels of HUWE1, we treated HeLa cells with IR (8Gy), doxorubicin (0.5 μg/ml) or etoposide (10 μM), respectively. The cellular protein level of HUWE1 was reduced following treatment with the DNA damage agents mentioned above (Figure 1A and B) and could be restored through treatment with the proteasome inhibitor MG132 (Figure 1C and D). These results reproduced a previous report of Khoronenkova & Dianov (22), and further suggest that HUWE1 was degraded through a ubiquitin-proteasome pathway in response to DNA damage. In order to identify the E3 ligases that may mediate HUWE1 ubiquitination and proteasomal degradation, we focused on those E3 ligases that are activated during the DDR process. Among them, Cullin 4B has been reported to be activated and plays a pivotal role in the DDR process (42). As the activity of CRLs is regulated through neddylation, and as DNA damage can induce neddylation (43), we asked whether CUL4B is the E3 ligase that is responsible for HUWE1 ubiquitination.

To test this hypothesis, the neddylation of CUL4B following DDR was examined. As shown in Figure 1A, a shifted band of CUL4B was detected after doxorubicin, etoposide or IR treatment, indicating CUL4B was modified under conditions of stress. Consistent with a modification by NEDD8, this modification of CUL4B was blocked by prior treatment of the cells with MLN4924, a NEDD8-activating enzyme inhibitor (Figure 1E). These results indicate that the DDR process can induce CUL4B neddylation. More importantly, the increased levels of CUL4B neddylation were inversely correlated with the decrease in HUWE1 levels (Figure 1A). These findings suggest that the activation of CRL4B may be responsible for the decreased protein levels of HUWE1. To confirm this, the neddylation pathway was blocked through the depletion of *UBC12* (a NEDD8 E2-conjugating enzyme), and its effect on HUWE1 was examined. As expected, the cellular levels of HUWE1 were reduced in control siRNA-treated cells after exposure to doxorubicin for 6 h while significantly stabilized upon depletion of *UBC12* (Figure 1F). Consistently, the inhibition of CUL4B neddylation through treatment with MLN4924 also rescued the reduction of HUWE1 levels following doxorubicin treatment (Figure 1G). Taken together, these results indicate that the inactivation of CUL4B blocks the degradation of HUWE1.

### CUL4B interacts with HUWE1 *in vivo*

**Figure 2.**
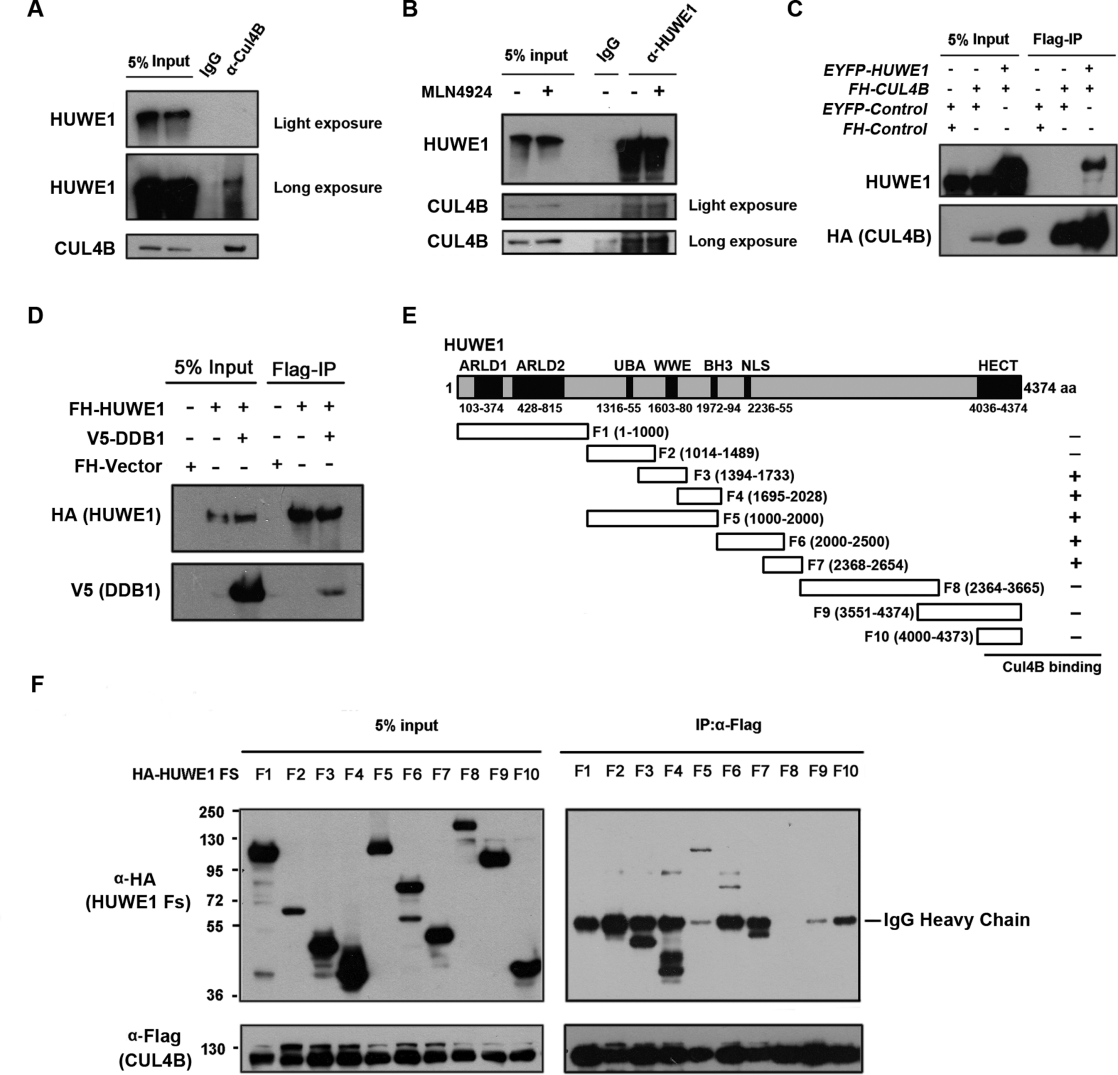
CUL4B interacts with HUWE1. (**A**) Endogenous CUL4B interacts with HUWE1. HeLa cell lysates were used for immunoprecipitation (IP) with CUL4B antibodies or normal IgG, the immunoprecipitates were then subjected to immunoblotting (IB) with the indicated antibodies. (**B**) Endogenous HUWE1 interacts with CUL4B. Endogenous HUWE1 was immunoprecipitated using a polyclonal antibody against HUWE1 from HeLa lysate treated with or without MLN4924, and CUL4B was detected in the HUWE1 immunoprecipitates. (**C**) Exogenous CUL4B interacts with HUWE1. 293T cells were cotransfected with *Flag-HA-CUL4B* (*FH-CUL4B*) and *EYFP-HUWE1* or *pEYFP-C1* empty plasmids. FH-CUL4B was pulled down using anti-FLAG M2 agarose and blotted with the indicated antibodies. (**D**) DDB1 interacts with HUWE1. 293T cells were transfected with *Flag-HA-HUWE1 (FH-HUWE1*), *FH-HUWE1* plus *V5-DDB1* or *pcDNA3.1-Flag-HA* empty plasmids. FH-HUWE1 was pulled down using anti-FLAG M2 agarose and blotted with the indicated antibodies. (**E**) Schematic representation of the *HUWE1* fragments fused with hemagglutinin (HA) tag. Plasmids encoding the indicated segments of *HUWE1* were subcloned into *pCMV-HA* plasmid to construct *pCMV-HA-HUWE1-F* series (F1-F10) for interaction mapping. (**F**) HUWE1-CUL4B interaction mapping. 293T cells were cotransfected with *pCMV-Tag2A-CUL4B* (for expression of Flag-tagged CUL4B) and *pCMV-HA-HUWE1-Fs*. Flag-tagged CUL4Bs were immunoprecipitated with anti-FLAG M2 agarose, followed by western blotting with the indicated anibodies. HUWE1 F3, F4 and F7 fragments were detected in Flag-CUL4B immunoprecipitates.

The correlation between the CUL4B ubiquitin E3 ligases and HUWE1 prompted us to investigate whether CUL4B interacts with HUWE1. Co-immunoprecipitation was performed with HeLa cell lysates, and HUWE1 was shown to be pulled down by CUL4B (Figure 2A). The reciprocal co-immunoprecipitation verified this interaction (Figure 2B). In addition, the endogenous HUWE1 was also pulled down by ectopic CUL4B which is immunoprecipitated from HeLa S3 cells that can stably express Flag-HA-tagged CUL4B (Supplementary Figure S1A). Likewise, the exogenous CUL4B bound to the exogenous HUWE1 (Figure 2C, Supplementary Figure S1B), which further confirms the interaction between CUL4B and HUWE1. To further determine whether the active state (neddylation) of CUL4B is essential for its interaction with HUWE1, we treated HeLa cells with MLN4924, and performed a co-immunoprecipitation assay. Our results showed that CUL4B was pulled down by HUWE1 regardless of the existence of MLN4924 (Figure 2B), suggesting that the neddylated state of CUL4B is not indispensable for its interaction with HUWE1. To further confirm the interaction between HUWE1 and CUL4B, we investigated the interaction of HUWE1 with DDB1. DDB1 is a main component of the CUL4B-ubiquitin-E3-ligase complex (CRL4B) that can mediate the interaction of substrates and the scaffolding of CUL4 (44). If HUWE1 is a substrate for CRL4B, then it should be able to bind to DDB1. As expected, HUWE1 was shown to pull down DDB1 (Figure 2D). Overall, these data suggest that HUWE1 interacts with the CRL4B complex.

We next mapped the regions of HUWE1 that are required for its interaction with CUL4B. Plasmids expressing a series of HA-tagged HUWE1 fragments were constructed (Figure 2E), and their ability to associate with the full-length form of CUL4B was assessed in 293T cells by co-immunoprecipitation following transfection. CUL4B primarily interacts with the F3 (WWE domain) and F4 (BH3 domain) domains of HUWE1, as well as F5, F6 and F7, suggesting that the WWE and BH3 domains, and the region comprising amino acids 2000–2654 of HUWE1 are essential to mediate its interaction with CUL4B. It has been demonstrated that the WWE domain mediates the protein–protein interactions, and the BH3 domain has been shown to be required for the interaction of MCL-1 with HUWE1. Interestingly, we found that F4 displayed high-molecular-weight band shifts (Figure 2F), implying that CUL4B induces the post-translational modification of HUWE1, and that this modification is most likely to be its ubiquitination.

### CUL4B controls the cellular levels of HUWE1

**Figure 3.**
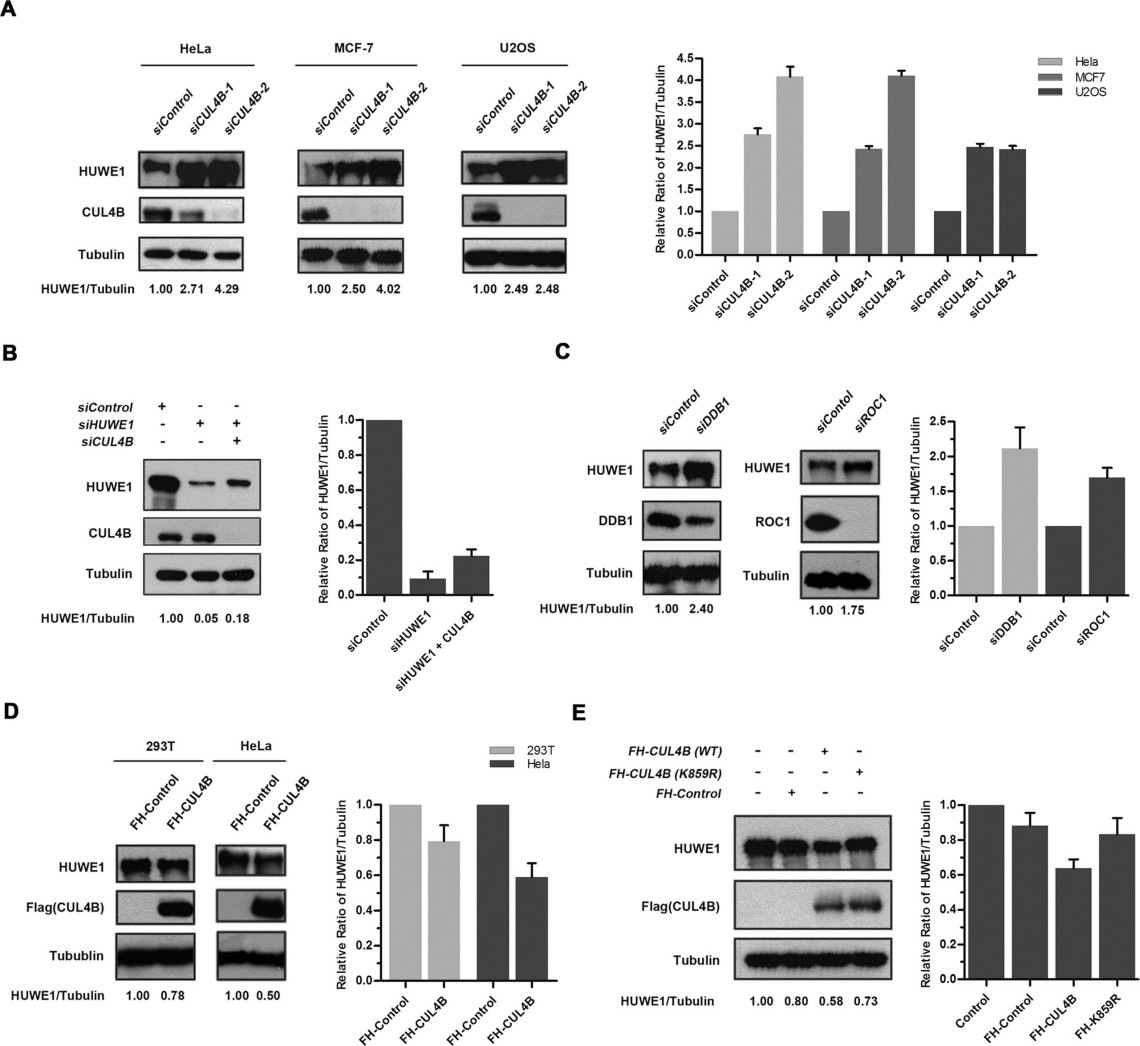
Cellular levels of HUWE1 are regulated by CUL4B E3 ligase. (**A**) HeLa, MCF-7 and U2OS cells were transfected with *CUL4B*-specific siRNA. Forty-eight hours after transfection, cells were lysed and analyzed by western blotting with the indicated antibodies. (**B**) HeLa cells were transfected with control, *HUWE1 or HUWE1* plus *CUL4B siRNA*. Forty-eight hours after transfection and cells were then lysed and analyzed by western blotting with the indicated antibodies. (**C**) HeLa cells were transfected with the indicated siRNA (*si-DDB1* and *si-ROC1*). Forty-eight hours after transfection, cells were lysed and analyzed by western blotting with the indicated antibodies. (**D**) 293T and Hela cells were transfected with either *pcDNA3.1-Flag-HA* or *pcDNA3.1-Flag-HA-CUL4B*, and analyzed by immunoblotting with the indicated antibodies. (**E**) 293T cells were transfected with *pcDNA3.1-Flag-HA*, *pcDNA3.1-Flag-HA-CUL4B* (WT) and *pcDNA3.1-Flag-HA-CUL4B* (K859R), respectively. Cells were harvested after 48 h transfection and analyzed by western blotting with the indicated antibodies. Right panels of A–E show quantification of the protein level of HUWE1 upon the indicated treatement. Columns, mean; Bars, ± S.D.

Ubiquitination by E3 ligases is often associated with degradation (45). To determine whether CUL4B controls the stability of the HUWE1 protein, *CUL4B* was depleted in HeLa cells and its effect on the stability of HUWE1 was assessed. As shown in Figure 3A, HUWE1 was significantly stabilized in *CUL4B* small interfering RNA (siRNA)-treated HeLa cells. Similar results were found using the MCF-7 and U2OS cell lines (Figure 3A). In addition, HUWE1 could be rescued through treatment with *CUL4B* siRNA in *HUWE1*-silenced cells (Figure 3B). Since DDB1 and ROC1 are indispensable for the activity of the CUL4B E3 ligase, we next investigated their influence on the protein levels of HUWE1, using siRNA against *DDB1* and *ROC1*. As expected, the treatment with either *DDB1* or *ROC1* siRNA led to a significant accumulation of HUWE1 protein when compared to controls (Figure 3C). Consistent with a role of CUL4B in the regulation of HUWE1 stability, the overexpression of *CUL4B* in 293T and HeLa cells reduced the protein levels of HUWE1 (Figure 3D). Furthermore, this effect was abolished in cells overexpressing the K859R mutant of *CUL4B*, in which the potential neddylation site lysine 859 was replaced by arginine and is presumably inactive (lysine 859 overlaps with the evolutionarily conserved neddylation site among other Cullins) (Figure 3E). Collectively, these results demonstrate that the CUL4B-DDB1-ROC1-E3-ligase complex negatively regulates the protein levels of HUWE1.

### CUL4B promotes the ubiquitination and degradation of HUWE1 *in vivo* and *in vitro*

**Figure 4.**
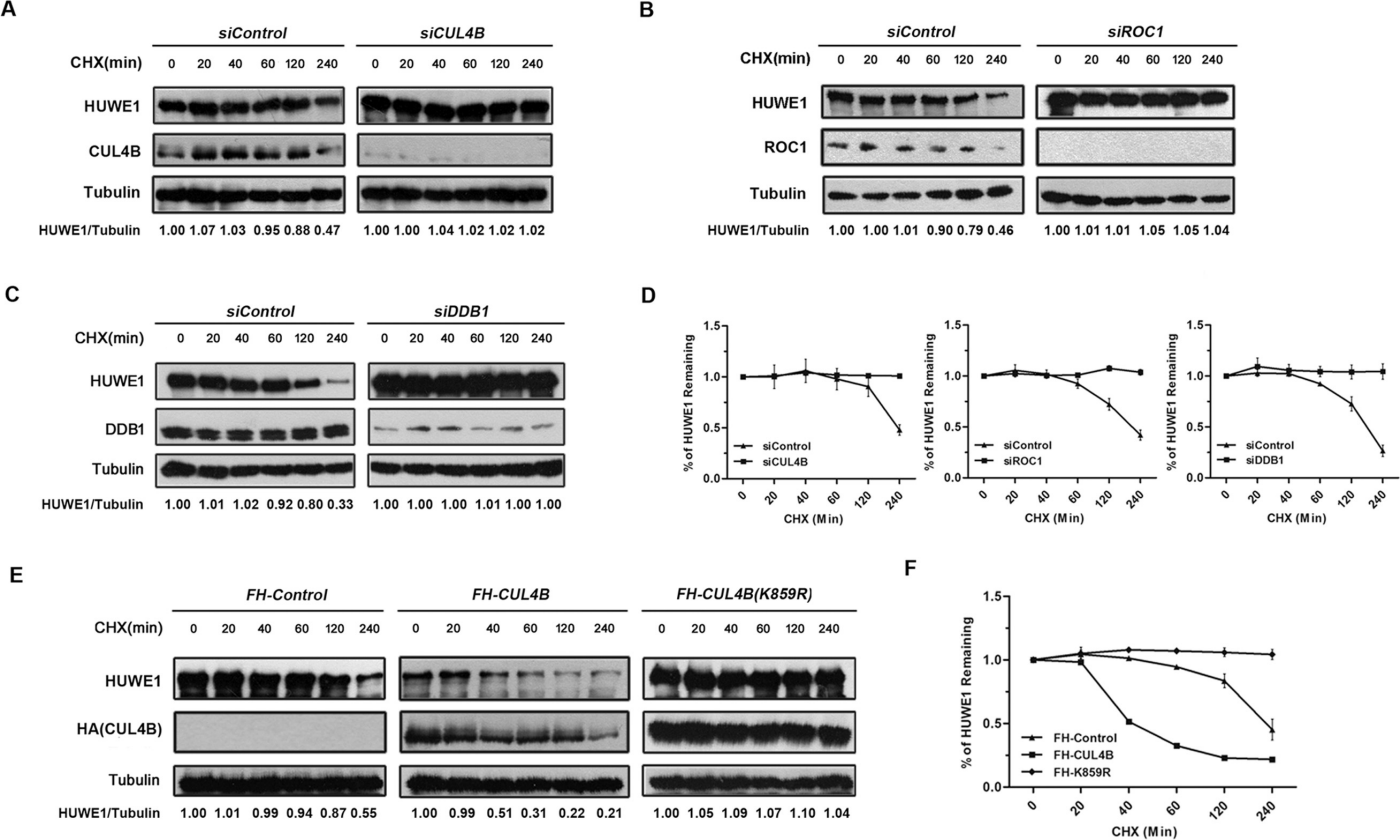
HUWE1 is stabilized upon RNAi-mediated depletion of CRL4B. (**A–C**) HUWE1 degradation is impaired in the cells depleted of *CUL4B*, *DDB1*or *ROC1*. HeLa cells were transfected with siRNA targeting *CUL4B*, *DDB1* or *ROC1*. Transfected cells were treated with cycloheximide (CHX, 100 μg/ml) to block *de novo* protein synthesis. Cells were harvested at the indicated time points after CHX treatment and protein levels were analyzed by western blotting with the indicated antibodies. (**D**) Quantification of HUWE1 as in (A–C) was plotted. (**E**) 293T cells were transfected with *pcDNA3.1-Flag-HA*, *pcDNA3.1-Flag-HA-CUL4B* wild type (WT) and *pcDNA3.1-Flag-HA-CUL4B* mutant (K859R) plasmids. Transfected cells were treated and analyzed as in (A–C). (**F**) Quantification of (E) was plotted.

Based on the findings that CUL4B negatively regulates HUWE1, we reasoned that the CRL4B may be the E3 ligase that is responsible for the ubiquitin- and proteasome-mediated degradation of HUWE1 in response to DNA damage. We first tested whether the stability of HUWE1 is dependent on the activity of CRL4B. Cycloheximide (CHX) was used to block *de novo* protein synthesis, and the half-life of HUWE1 was assessed in cells that had been transfected with either a negative control or with *CUL4B* siRNA. As shown in Figure 4A–D, the siRNA silencing of *CUL4B*, *DDB1* or *ROC1* significantly prolonged the half-life of HUWE1, suggesting that the integrity of the DDB1-CUL4B-ROC1-E3-ligase complex is essential for the degradation of HUWE1. Consistently, the overexpression of *Flag-HA-CUL4B* dramatically accelerated the degradation of HUWE1, whereas the overexpression of the *Flag-HA-CUL4B K859R* mutant caused a remarkable stabilization, and prolonged the half-life of HUWE1 (Figure 4E and F). These results suggest that the CUL4B ubiquitin E3 ligase effectively promotes the degradation of HUWE1 *in vivo* through the ubiquitin-proteasome pathway.

**Figure 5.**
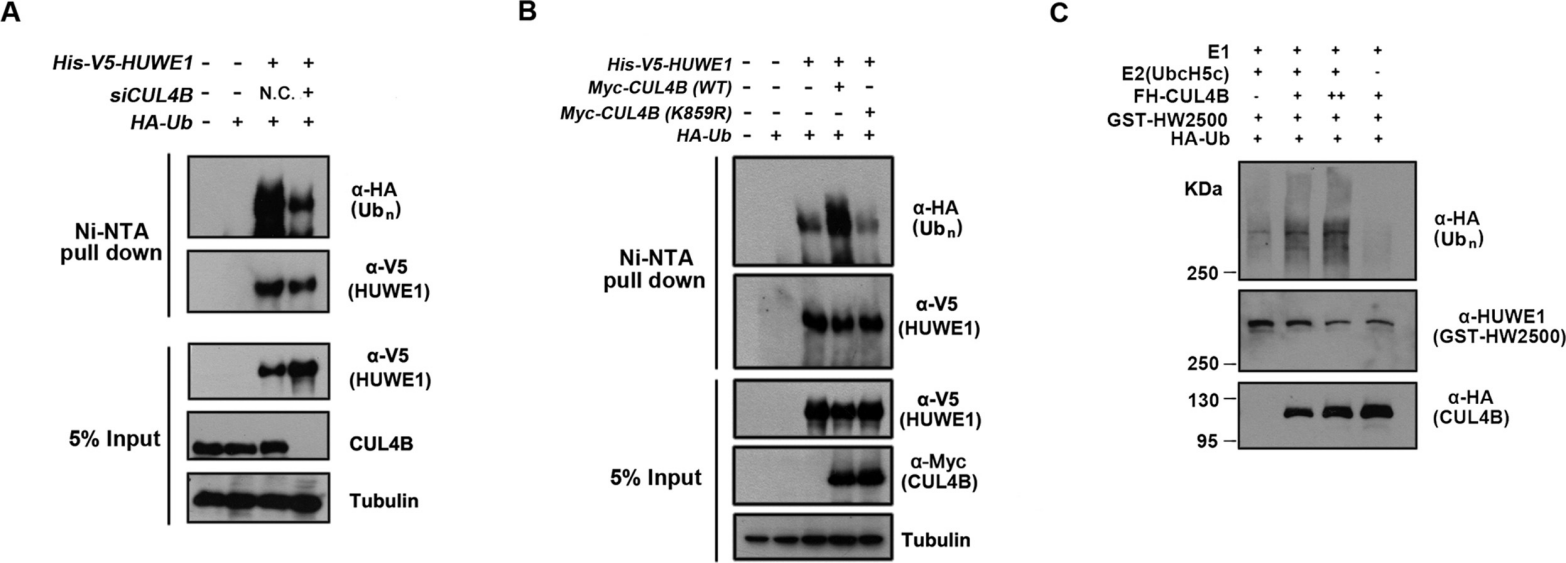
CUL4B E3 ligase targets HUWE1 for ubiquitination *in vivo* and *in vitro*. (**A**) 293T cells transfected with indicated plasmids and siRNA targeting *CUL4B* were lysed and subjected to Ni-NTA pull-down under denaturing condition. The resulting pull-downs were subjected to immunoblotting with antibodies against HA or V5. A portion of the input lysates was also subjected directly to immunoblotting analysis with indicated antibodies. (**B**) 293T cells transfected with indicated plasmids were lysed and subjected to Ni-NTA pull-down and immunoblotting as in (A). (**C**) *In vitro* ubiquitination assay. Recombinant GST-HW2500 protein was incubated with ATP regenerating buffer, E1, E2 (UbcH5c), recombinant Flag-HA-CUL4B and HA-ubiquitin in a 30-μl reaction volume for 1.5 h at 30°C. The reaction mixtures were analyzed by western blotting with HA and HUWE1 antibodies.

Next, we examined the role of CRL4B on the ubiquitination of HUWE1 *in vivo*. 293T cells were transfected with His-V5-tagged HUWE1 and with HA-tagged Ub plasmids, and were simultaneously depleted of endogenous *CUL4B* through siRNA interference (control or *siCUL4B*). An immunoblotting analysis of the Ni-NTA pull-down showed that the ubiquitination of HUWE1 was impaired when compared to controls (Figure 5A). Conversely, the overexpression of the wild-type form of *Myc-CUL4B*, but not that of the *Myc-K859R* mutant, led to increased ubiquitination of HUWE1 (Figure 5B). As we had demonstrated that the CUL4B complex promotes the polyubiquitination of HUWE1 *in vivo*, we proceeded to perform *in vitro* ubiquitination assays. To produce the CUL4B complex, we transiently expressed *Flag-HA-CUL4B* in 293T cells. *Flag-HA-CUL4B* was highly expressed in 293T cells (Supplementary Figure S1C), and the CUL4B complex was successfully purified using an anti-FLAG-M2 agarose gel (Supplementary Figure S2A). Based on the mapping assay (Figure 2E and F), we inferred that the ubiquitination site might be located somewhere between amino acids1-2500 of HUWE1. We therefore constructed a GST-tagged *HUWE1-1-2500* (*GST-HW2500*) plasmid for the generation of the baculovirus. GST-HW2500 was successfully expressed and purified from SF9 cells infected with the *GST-HW2500* baculovirus (Supplementary Figures S1D and S2B). An *in vitro* ubiquitination assay was performed, and the results showed that CUL4B promotes the ubiqutination of HUWE1 (Figure 5C, lanes 2 and 3). Taken together, these data demonstrate that the CUL4B-E3-ligase complex targets HUWE1 for ubiquitination.

### The inhibition of CUL4B sensitizes cells to DNA damage-induced apoptosis, through the upregulation of HUWE1

**Figure 6.**
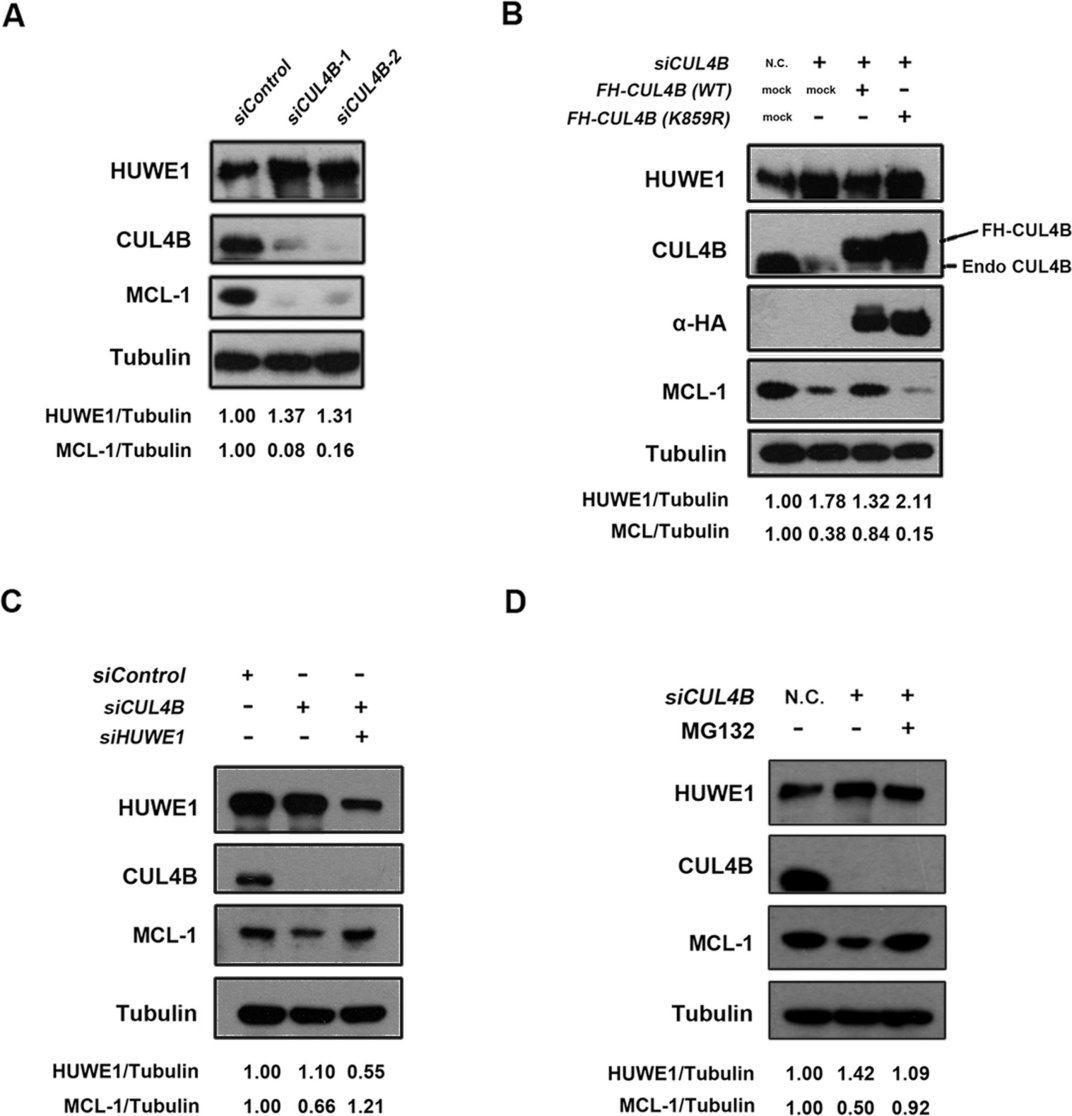
CUL4B controls MCL-1 protein level through regulation of HUWE1. (**A**) MCL-1 is downregulated upon *CUL4B* depletion. HeLa cells were transfected with the indicated siRNA for 48 h, lysed and analyzed by western blotting with the indicated antibodies. (**B**) Ectopic expression of wild type, but not the neddylation-deficient *CUL4B* K859R mutant, rescued *CUL4B* RNAi-mediated downregulation of MCL-1. HeLa cells were cotransfected with *pcDNA3.1-Flag-HA-CUL4B* (*WT*), *pcDNA3.1-Flag-HA-CUL4B* (*K859R*) or empty plasmids, and siRNA targeting 3’UTR *of CUL4B* gene, which can deplete endogenous but not ectopic expressed CUL4B. Forty-eight hours after transfection, cells were lysed and analyzed by western blotting with the indicated antibodies. (**C**) CUL4B mediated-downregulation of MCL-1 is rescued by concomitant depletion of *HUWE1*. U2OS cells were transfected with *CUL4B*, *HUWE1* or *CUL4B* plus *HUWE1* siRNA for 48 h, respectively. Cells were lysed and analyzed by western blotting with the indicated antibodies. (**D**) *CUL4B* RNAi-mediated downregulation of MCL-1 is proteasome-dependent. HeLa cells were transfected with *CUL4B* siRNA and treated either with or without proteasome inhibitor MG-132 (20 μM) for 4 h. Cells were then lysed and analyzed by western blotting with the indicated antibodies.

MCL-1 is a member of the anti-apoptotic BCL-2 family that regulates the mitochondrial response to apoptotic stimuli and that protects mitochondrial integrity. HUWE1 has been reported to contain a BH3 domain that can mediate the ubiquitination and degradation of MCL-1. The elimination of HUWE1 expression through RNA interference stabilizes the MCL-1 protein, resulting in attenuation of the apoptosis induced by DNA damage agents (8). As CUL4B can promote the degradation of HUWE1, we therefore asked whether CUL4B can stabilize MCL-1 through the downregulation of HUWE1. As expected, the overexpression of *Flag-HA-CUL4B* increased the protein levels of MCL-1 (Supplementary Figure S1E). Similarly, the depletion of *CUL4B* resulted in a significant decrease of MCL-1 (Figure 6A), which is consistent with the increased protein levels of HUWE1 caused by the inhibition of *CUL4B*. In addition, the alteration of both the HUWE1 and MCL-1 proteins resulting from *CUL4B* depletion could be rescued through the co-expression of the wild-type, but not the K859R mutant form of the *Flag-HA-tagged CUL4B* plasmid (Figure 6B). More importantly, the decrease in MCL-1 caused by *CUL4B* knockdown was offset by the simultaneous depletion of *HUWE1* (*CUL4B/HUWE1* siRNA) (Figure 6C). These results suggest that the reduction of MCL-1 in *CUL4B*-depleted cells was indeed mediated by the upregulation of HUWE1. We thus inferred that the proteasomal degradation of MCL-1 could be enhanced in the *CUL4B*-depleted cells. To this end, HeLa cells that had been transfected either with a *CUL4B* or with a control siRNA were treated with MG132, and MCL-1 expression was examined. As expected, the reduction of the MCL-1 protein was blocked following treatment with MG132 (Figure 6D). Taken together, these data demonstrate that CRL4B is a critical regulator of HUWE1-mediated apoptosis in response to DNA damage stimuli.

**Figure 7.**
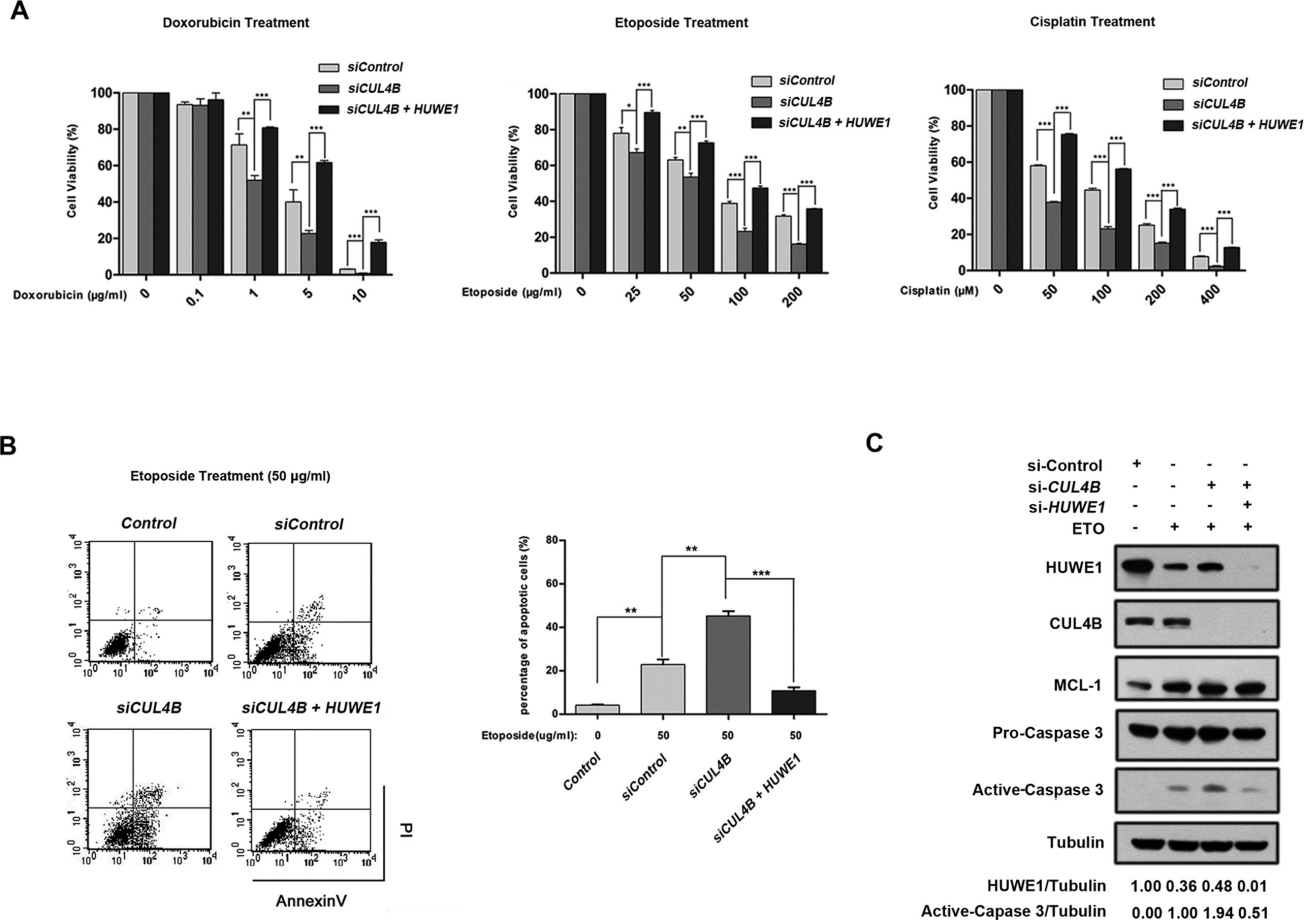
Silencing of *CUL4B* sensitizes cells to the DNA damage-induced apoptosis. (**A**) U2OS cells transfected with the control, *CUL4B* or *CUL4B* plus *HUWE1* siRNA for 48 h were treated with doxorubicin (Dox), etoposide (Eto) and cisplatin (Cis) at the indicated concentration for 24 h. The survival rate of cells was assessed by MTT assay. Data were represented as mean ± S.D. All of the assays were performed in triplicate. (**B**) U2OS cells were transfected with control, *CUL4B or CUL4B* plus *HUWE1* for 48 h, followed by etoposide treatment at 50 μg/ml for another 24 h. Cells were harvested, labeled with Annexin V/PI and analyzed by flow cytometry. Left panel shows a typical flow cytometry plot. Right panel represents histograms of all data. Data were represented as mean ± S.D. All assays were performed in triplicate. Columns, mean; Bars, ±S.D. (*P < 0.05; **P < 0.01; ***P < 0.001, t-test, two-sided). (**C**) U2OS cells were treated as in (B) and analyzed by western blotting with the indicated antibodies.

To further investigate the functional link between CUL4B and HUWE1 as well as its biological significance, we performed a sensitivity assay following the treatment of DNA damage agents. U2OS cells that had been transfected with either *CUL4B* or *CUL4B*+*HUWE1* siRNA were treated with doxorubicin, etoposide and cisplatin at different concentrations. An MTT assay showed that the survival rate was decreased in *CUL4B*-silenced cells compared to that of the control cells, whereas in *CUL4B* and *HUWE1* double-silenced cells, the survival rate was restored to a higher level (Figure 7A). To confirm that CUL4B inhibition can enhance apoptosis induced by DNA damage agents, U2OS cells that had been transfected with *CUL4B* or *CUL4B + HUWE1* siRNA were treated with etoposide at the same concentration, and apoptotic cells were analyzed using flow cytometry. The results indicated that *CUL4B* depletion resulted in an increase in the apoptotic rate when compared with the control group, whereas the concomitant depletion of both *CUL4B* and *HUWE1* caused a substantial decrease in the apoptotic rate (Figure 7B). To further confirm the apoptosis induced by *CUL4B* depletion, activation of caspase-3 was examined by immunoblotting. As expected, cleaved caspase-3 was induced in cells treated with the DNA damage agents, and enhanced when *CUL4B* was depleted by RNAi. Furthermore, this effect of *CUL4B* depletion on caspase-3 induction was attenuated by simultaneous knockdown of HUWE1 (Figure 7C). Taken together, these results suggest that the CUL4B-mediated regulation of HUWE1 plays an essential role in the modulation of apoptosis and in the sensitivity to DNA damage agents.

## DISCUSSION

E3 ligases are key effectors and regulators involved in multiple cellular processes in response to DNA damage (46,47). In this study, we showed that the DNA damage-induced downregulation of HUWE1 was dependent on CRL4B. CRL4B ubiquitinated and targeted HUWE1 for proteasomal degradation. Consistent with a role of CRL4B in the regulation of HUWE1, the depletion or the inactivation of CRL4B resulted in increased degradation of MCL-1, a known HUWE1 substrate involved in anti-apoptosis, and therefore sensitized the cells to DNA damage-induced apoptosis.

Two members of the CUL4 family, CUL4A and CUL4B, exist in mammals (including humans), and have been reported to share overlapping functions through targeting the same substrate, such as CDT1, cyclin E, p21, and histone H3 and H4, for ubiquitination (42,48,49). However, CUL4B is structurally different from CUL4A in that it contains an extended N terminus. In addition, the respective tissue-specific expression patterns of CUL4A and CUL4B also differ, suggesting that CUL4B has a distinct function (26,50). Several CRL4B-specific substrates, including Topo I, PrxIII, WDR5, ER-α and Jab1, have recently been identified (28,30,51–53). Here, we identify HUWE1 as a novel and specific substrate of the CUL4B ubiquitin ligase during the DDR process. This finding reveals a novel function of CUL4B and provides new insight into the mechanism underlying HUWE1 regulation during the process of DDR.

HUWE1 is a HECT-containing ubiquitin E3 ligase that can mediate the ubiquitination and the degradation of multiple key regulators such as p53, MCL-1, c-MYC and CDC6, which are involved in the cell cycle and in checkpoints, apoptosis, DNA replication, as well as DNA damage repair. Under physiological conditions, substantial levels of HUWE1 are maintained, presumably through the targeting of p53 for proteasomal degradation, thereby keeping the steady state of p53 at a low level. In addition, HUWE1 has also been shown to be required for the promotion of cell survival and tumorigenesis, through the K63-linkage ubiquitination of c-MYC and hence its activation in cancer cells (15). Recently, we demonstrated that HUWE1 plays an essential role in regulating the stability of BRCA1, a critical tumor suppressor involved in breast cancer tumorigenesis (13). These findings highlight the crucial role of HUWE1 in promoting the proliferation and the survival of unstressed cells, through the negative regulation of tumor suppressors such as p53 and BRCA1, and/or the positive regulation of onco-proteins such as c-MYC. In genotoxic conditions, however, a different scenario regarding the function of HUWE1 is observed. HUWE1 seems to target key players that are involved in apoptosis, DNA replication, and DNA damage repair for ubiquitination and degradation. For example, MCL-1 and CDC6 (but not p53 and BRCA1) were ubiquitinated and degraded, leading to apoptosis and stalled DNA replication. Therefore, the function of HUWE1 is highly regulated in the DDR pathway. Despite its role in DNA damage, we and others (17,18,22) also showed that DNA damage can also induce the inhibition of HUWE1 activity. However, the mechanism underlying HUWE1 regulation in DDR remains elusive. Previous studies reported that the self-ubiquitination of HUWE1 is responsible for its degradation following DNA damage (17,22). The inactivation of USP4 and USP7, two deubiquitinating(DUB) enzymes that can deubiquitinate HUWE1, leads to low protein levels of HUWE1, indicating that DUBs are involved in the regulation of HUWE1 in response to DNA damage. Apart from these DUBs, E3 ligases may also play a role in the regulation of HUWE1 stability. Consistent with this hypothesis, we showed that CUL4B can interact with HUWE1 and mediate its ubiquitination and degradation. This study reveals a novel mechanism through which HUWE1 is regulated following DNA damage.

In this study, we found that HUWE1 and CUL4B reciprocally interacted with each other. However, their interaction was pretty weak (Figure 2A|C). A speculative explanation is that they might transiently interact with each other, or in a ‘hit and run’ mechanism, which is employed by many enzymes and their substrates (54). Despite their weak interaction, the integrity of the CUL4B-DDB1-ROC1-E3-ligase complex was found to be indispensable for the ubiquitination and degradation of HUWE1. The CUL4B-DDB1-ROC1 complex can either interact with its substrates directly through DDB1 or indirectly through a family of adaptors called the DWD proteins (55,56), it is unclear whether the HUWE1 protein itself directly binds to DDB1 or is indirectly bridged by an unknown adaptor protein yet to be identified.

CUL4B has been shown to participate in the DNA damage repair pathway or in histone ubiquitination (48,57,58), and was found to be aberrantly expressed in a variety of cancers types, including breast cancer (59,60), and correlated with cancer progression and pathogenesis (61). These data, together with the fact that CRL4 is activated through neddylation in the DDR pathway, suggest a potential strategy for anti-tumor therapy, through the inhibition of CRL4 using MLN4924, an inhibitor of the NEDD8-activating enzyme that can effectively inhibit the neddylation of the Cullin family proteins (62). The inactivation of CRL E3 ligases results in the accumulation of its substrates, and thereby leads to enhanced apoptosis in response to DNA damage, and MLN4924 has been reportedly applied in cancer therapy (62–65). In our study, we found that the depletion or the inactivation of CUL4B resulted in the accumulation of HUWE1, and thereby accelerated the degradation of its substrate, MCL-1, rendering cancer cells sensitive to DNA damage agents. Our findings provide novel insights for a cancer therapy strategy through the interference with CRL4B.

## SUPPLEMENTARY DATA

Supplementary Data are available at NAR Online.

SUPPLEMENTARY DATA

## References

[1] Ciccia A., Elledge S.J. (2010). The DNA damage response: making it safe to play with knives. Mol. Cell.

[2] Harper J.W., Elledge S.J. (2007). The DNA damage response: ten years after. Mol. Cell.

[3] Silverman J.S., Skaar J.R., Pagano M. (2012). SCF ubiquitin ligases in the maintenance of genome stability. Trends Biochem. Sci..

[4] Messick T.E., Greenberg R.A. (2009). The ubiquitin landscape at DNA double-strand breaks. J. Cell Biol..

[5] Wrighton K.H. (2014). DNA damage response: a ligase makes sense of DNA damage. Nat. Rev. Mol. Cell Biol..

[6] Pickart C.M. (2004). Back to the future with ubiquitin. Cell.

[7] Bhoj V.G., Chen Z.J. (2009). Ubiquitylation in innate and adaptive immunity. Nature.

[8] Zhong Q., Gao W., Du F, Wang X. (2005). Mule/ARF-BP1, a BH3-only E3 ubiquitin ligase, catalyzes the polyubiquitination of Mcl-1 and regulates apoptosis. Cell.

[9] Parsons J.L., Tait P.S., Finch D., Dianova I.I., Edelmann M.J., Khoronenkova S.V., Kessler B.M., Sharma R.A., McKenna W.G., Dianov G.L. (2009). Ubiquitin ligase ARF-BP1/Mule modulates base excision repair. EMBO J..

[10] Hall J.R., Kow E., Nevis K.R., Lu C.K., Luce K.S., Zhong Q., Cook J.G. (2007). Cdc6 stability is regulated by the Huwe1 ubiquitin ligase after DNA damage. Mol. Biol. Cell.

[11] Herold S., Hock A., Herkert B., Berns K., Mullenders J., Beijersbergen R., Bernards R., Eilers M. (2008). Miz1 and HectH9 regulate the stability of the checkpoint protein, TopBP1. EMBO J..

[12] Markkanen E., van Loon B., Ferrari E., Parsons J.L., Dianov G.L., Hubscher U. (2012). Regulation of oxidative DNA damage repair by DNA polymerase lambda and MutYH by cross-talk of phosphorylation and ubiquitination. Proc. Natl Acad. Sci. U. S. A..

[13] Wang X., Lu G., Li L., Yi J., Yan K., Wang Y., Zhu B., Kuang J., Lin M., Zhang S. (2014). HUWE1 interacts with BRCA1 and promotes its degradation in the ubiquitin-proteasome pathway. Biochem. Biophys. Res. Commun..

[14] Zhao X., Heng J.I., Guardavaccaro D., Jiang R., Pagano M., Guillemot F., Iavarone A., Lasorella A. (2008). The HECT-domain ubiquitin ligase Huwe1 controls neural differentiation and proliferation by destabilizing the N-Myconcoprotein. Nat. Cell Biol..

[15] Adhikary S., Marinoni F., Hock A., Hulleman E., Popov N., Beier R., Bernard S., Quarto M., Capra M., Goettig S. (2005). The ubiquitin ligase HectH9 regulates transcriptional activation by Myc and is essential for tumor cell proliferation. Cell.

[16] Yang Y., Do H., Tian X., Zhang C., Liu X., Dada L.A., Sznajder J.I., Liu J. (2010). E3 ubiquitin ligase Mule ubiquitinates Miz1 and is required for TNFalpha-induced JNK activation. Proc. Natl Acad. Sci. U. S. A..

[17] Zhang X., Berger F.G., Yang J., Lu X. (2011). USP4 inhibits p53 through deubiquitinating and stabilizing ARF-BP1. EMBO J..

[18] Chen D., Kon N., Li M., Zhang W., Qin J., Gu W. (2005). ARF-BP1/Mule is a critical mediator of the ARF tumor suppressor. Cell.

[19] Leboucher G.P., Tsai Y.C., Yang M., Shaw K.C., Zhou M., Veenstra T.D., Glickman M.H., Weissman A.M. (2012). Stress-induced phosphorylation and proteasomal degradation of mitofusin 2 facilitates mitochondrial fragmentation and apoptosis. Mol. Cell.

[20] Zhou X., Li T.T., Feng X., Hsiang E., Xiong Y., Guan K.L., Lei Q.Y. (2012). Targeted polyubiquitylation of RASSF1C by the Mule and SCFbeta-TrCP ligases in response to DNA damage. Biochem. J..

[21] Zhang J., Kan S., Huang B., Hao Z., Mak T.W., Zhong Q. (2011). Mule determines the apoptotic response to HDAC inhibitors by targeted ubiquitination and destruction of HDAC2. Genes Dev..

[22] Khoronenkova S.V., Dianov G.L. (2013). USP7S-dependent inactivation of Mule regulates DNA damage signalling and repair. Nucleic Acids Res..

[23] Sherr C.J. (2001). The INK4a/ARF network in tumour suppression. Nat. Rev. Mol. Cell Biol..

[24] Kamijo T., van de Kamp E., Chong M.J., Zindy F., Diehl J.A., Sherr C.J., McKinnon P.J. (1999). Loss of the ARF tumor suppressor reverses premature replicative arrest but not radiation hypersensitivity arising from disabled atm function. Cancer Res..

[25] Petroski M.D., Deshaies R.J. (2005). Function and regulation of cullin-RING ubiquitin ligases. Nat. Rev. Mol. Cell Biol..

[26] Jackson S., Xiong Y. (2009). CRL4s: the CUL4-RING E3 ubiquitin ligases. Trends Biochem. Sci..

[27] Liu L., Lee S., Zhang J., Peters S.B., Hannah J., Zhang Y., Yin Y., Koff A., Ma L., Zhou P. (2009). CUL4A abrogation augments DNA damage response and protection against skin carcinogenesis. Mol. Cell.

[28] Nakagawa T., Xiong Y. (2011). X-linked mental retardation gene CUL4B targets ubiquitylation of H3K4 methyltransferase component WDR5 and regulates neuronal gene expression. Mol. Cell.

[29] Zou Y., Mi J., Cui J., Lu D., Zhang X., Guo C., Gao G., Liu Q., Chen B., Shao C. (2009). Characterization of nuclear localization signal in the N terminus of CUL4B and its essential role in cyclin E degradation and cell cycle progression. J. Biol. Chem..

[30] Ohtake F., Baba A., Takada I., Okada M., Iwasaki K., Miki H., Takahashi S., Kouzmenko A., Nohara K., Chiba T. (2007). Dioxin receptor is a ligand-dependent E3 ubiquitin ligase. Nature.

[31] Isidor B., Pichon O., Baron S, David A., Le Caignec C. (2010). Deletion of the CUL4B gene in a boy with mental retardation, minor facial anomalies, short stature, hypogonadism, and ataxia. Am. J. Med. Genet. A.

[32] Badura-Stronka M., Jamsheer A., Materna-Kiryluk A., Sowinska A., Kiryluk K., Budny B., Latos-Bielenska A. (2010). A novel nonsense mutation in CUL4B gene in three brothers with X-linked mental retardation syndrome. Clin. Genet..

[33] Pan Z.Q., Kentsis A., Dias D.C., Yamoah K., Wu K. (2004). Nedd8 on cullin: building an expressway to protein destruction. Oncogene.

[34] Dye B.T., Schulman B.A. (2007). Structural mechanisms underlying posttranslational modification by ubiquitin-like proteins. Annu. Rev. Biophys. Biomol. Struct..

[35] Yeh E.T., Gong L., Kamitani T. (2000). Ubiquitin-like proteins: new wines in new bottles. Gene.

[36] Rabut G., Le Dez G., Verma R., Makhnevych T., Knebel A., Kurz T., Boone C., Deshaies R.J., Peter M. (2011). The TFIIH subunit Tfb3 regulates cullinneddylation. Mol. Cell.

[37] Kamura T., Conrad M.N., Yan Q., Conaway R.C., Conaway J.W. (1999). The Rbx1 subunit of SCF and VHL E3 ubiquitin ligase activates Rub1 modification of cullins Cdc53 and Cul2. Genes Dev..

[38] Kurz T., Ozlu N., Rudolf F., O'Rourke S.M., Luke B., Hofmann K., Hyman A.A., Bowerman B., Peter M. (2005). The conserved protein DCN-1/Dcn1p is required for cullinneddylation in C. elegans and S. cerevisiae. Nature.

[39] Meyer-Schaller N., Chou Y.C., Sumara I., Martin D.D., Kurz T., Katheder N., Hofmann K., Berthiaume L.G., Sicheri F., Peter M. (2009). The human Dcn1-like protein DCNL3 promotes Cul3 neddylation at membranes. Proc. Natl Acad. Sci. U. S. A..

[40] Kurz T., Chou Y.C., Willems A.R., Meyer-Schaller N., Hecht M.L., Tyers M., Peter M., Sicheri F. (2008). Dcn1 functions as a scaffold-type E3 ligase for cullinneddylation. Mol. Cell.

[41] Ma T., Shi T., Huang J., Wu L., Hu F., He P., Deng W., Gao P., Zhang Y., Song Q. (2008). DCUN1D3, a novel UVC-responsive gene that is involved in cell cycle progression and cell growth. Cancer Sci..

[42] Hu J., McCall C.M., Ohta T., Xiong Y. (2004). Targeted ubiquitination of CDT1 by the DDB1-CUL4A-ROC1 ligase in response to DNA damage. Nat. Cell Biol..

[43] Ma T., Chen Y., Zhang F., Yang C.Y., Wang S., Yu X. (2013). RNF111-dependent neddylation activates DNA damage-induced ubiquitination. Mol. Cell.

[44] Lee J., Zhou P. (2007). DCAFs, the missing link of the CUL4-DDB1 ubiquitin ligase. Mol. Cell.

[45] Kravtsova-Ivantsiv Y., Ciechanover A. (2011). Ubiquitination and degradation of proteins. Methods Mol. Biol..

[46] Huang T.T., D'Andrea A.D. (2006). Regulation of DNA repair by ubiquitylation. Nat. Rev. Mol. Cell Biol..

[47] Al-Hakim A., Escribano-Diaz C., Landry M.C., O'Donnell L., Panier S., Szilard R.K., Durocher D. (2010). The ubiquitous role of ubiquitin in the DNA damage response. DNA Repair (Amst).

[48] Wang H., Zhai L., Xu J., Joo H.Y., Jackson S., Erdjument-Bromage H., Tempst P., Xiong Y., Zhang Y. (2006). Histone H3 and H4 ubiquitylation by the CUL4-DDB-ROC1 ubiquitin ligase facilitates cellular response to DNA damage. Mol. Cell.

[49] Higa L.A., Yang X., Zheng J., Banks D., Wu M., Ghosh P., Sun H., Zhang H. (2006). Involvement of CUL4 ubiquitin E3 ligases in regulating CDK inhibitors Dacapo/p27Kip1 and cyclin E degradation. Cell Cycle.

[50] Hori T., Osaka F., Chiba T., Miyamoto C., Okabayashi K., Shimbara N., Kato S., Tanaka K. (1999). Covalent modification of all members of human cullin family proteins by NEDD8. Oncogene.

[51] Kerzendorfer C., Whibley A., Carpenter G., Outwin E., Chiang S.C., Turner G., Schwartz C., El-Khamisy S., Raymond F.L., O'Driscoll M. (2010). Mutations in Cullin 4B result in a human syndrome associated with increased camptothecin-induced topoisomerase I-dependent DNA breaks. Hum. Mol. Genet..

[52] He F., Lu D., Jiang B., Wang Y., Liu Q., Liu Q., Shao C., Li X., Gong Y. (2013). X-linked intellectual disability gene CUL4B targets Jab1/CSN5 for degradation and regulates bone morphogenetic protein signaling. Biochim. Biophys. Acta.

[53] Li X., Lu D., He F., Zhou H., Liu Q., Wang Y., Shao C., Gong Y. (2011). Cullin 4B protein ubiquitin ligase targets peroxiredoxin III for degradation. J. Biol. Chem..

[54] Deffenbaugh A.E., Scaglione K.M., Zhang L., Moore J.M., Buranda T., Sklar L.A., Skowyra D. (2003). Release of ubiquitin-charged Cdc34-S - Ub from the RING domain is essential for ubiquitination of the SCF(Cdc4)-bound substrate Sic1. Cell.

[55] Higa L.A., Wu M., Ye T., Kobayashi R., Sun H., Zhang H. (2006). CUL4-DDB1 ubiquitin ligase interacts with multiple WD40-repeat proteins and regulates histone methylation. Nat. Cell Biol..

[56] Angers S., Li T., Yi X., MacCoss M.J., Moon R.T., Zheng N. (2006). Molecular architecture and assembly of the DDB1-CUL4A ubiquitin ligase machinery. Nature.

[57] Sugasawa K., Okuda Y., Saijo M., Nishi R., Matsuda N., Chu G., Mori T., Iwai S., Tanaka K., Tanaka K. (2005). UV-induced ubiquitylation of XPC protein mediated by UV-DDB-ubiquitin ligase complex. Cell.

[58] Hu H., Yang Y., Ji Q., Zhao W., Jiang B., Liu R., Yuan J., Liu Q., Li X., Zou Y. (2012). CRL4B catalyzes H2AK119 monoubiquitination and coordinates with PRC2 to promote tumorigenesis. Cancer Cell.

[59] Chen L.C., Manjeshwar S., Lu Y., Moore D., Ljung B.M., Kuo W.L., Dairkee S.H., Wernick M., Collins C., Smith H.S. (1998). The human homologue for the Caenorhabditiselegans cul-4 gene is amplified and overexpressed in primary breast cancers. Cancer Res..

[60] Pan W.W., Zhou J.J., Yu C., Xu Y., Guo L.J., Zhang H.Y., Zhou D., Song F.Z., Fan H.Y. (2013). Ubiquitin E3 ligase CRL4(CDT2/DCAF2) as a potential chemotherapeutic target for ovarian surface epithelial cancer. J. Biol. Chem..

[61] Jiang T., Tang H.M., Wu Z.H., Chen J., Lu S., Zhou C.Z., Yan D.W., Peng Z.H. (2013). Cullin 4B is a novel prognostic marker that correlates with colon cancer progression and pathogenesis. Med. Oncol..

[62] Soucy T.A., Smith P.G., Milhollen M.A., Berger A.J., Gavin J.M., Adhikari S., Brownell J.E., Burke K.E., Cardin D.P., Critchley S. (2009). An inhibitor of NEDD8-activating enzyme as a new approach to treat cancer. Nature.

[63] Yang D., Tan M., Wang G., Sun Y. (2012). The p21-dependent radiosensitization of human breast cancer cells by MLN4924, an investigational inhibitor of NEDD8 activating enzyme. PLoS One.

[64] Soucy T.A., Dick L.R., Smith P.G., Milhollen M.A., Brownell J.E. (2010). The NEDD8 conjugation pathway and its relevance in cancer biology and therapy. Genes Cancer.

[65] Zhao Y., Xiong X., Jia L., Sun Y. (2012). Targeting Cullin-RING ligases by MLN4924 induces autophagy via modulating the HIF1-REDD1-TSC1-mTORC1-DEPTOR axis. Cell Death Dis..

