# Supplementary material for: DNA damage-induced activation of CUL4B targets HUWE1 for proteasomal degradation
Source: Nucleic Acids Res. 2015 Apr 16;43(9):4579–90. doi: 10.1093/nar/gkv325 (PMC4482080; doi:10.1093/nar/gkv325)
Supplement: SUPPLEMENTARY DATA [file supp_43_9_4579__index.html]

DNA damage-induced activation of CUL4B targets HUWE1 for proteasomal degradation — SUPPLEMENTARY DATA 

# DNA damage-induced activation of CUL4B targets HUWE1 for proteasomal degradation

## SUPPLEMENTARY DATA

**Files in this Data Supplement:**

- SUPPLEMENTARY DATA
