# Supplementary material for: DNA damage-induced activation of CUL4B targets HUWE1 for proteasomal degradation
Source: Nucleic Acids Res. 2015 Apr 16;43(9):4579–90. doi: 10.1093/nar/gkv325 (PMC4482080; doi:10.1093/nar/gkv325)
Supplement: SUPPLEMENTARY DATA [file supp_gkv325_nar-03455-d-2014-File009.pdf]

SUPPORTING INFORMATION

MATERIALS AND METHODS

Plasmids

All Huwe1-related plasmids in this study were constructed by PCR using *pFastBac-Mule* plasmid as templates. For construction of *pcDNA3.1-Flag-HA-HUWE1*, a Flag-HA double tag was introduced into *pcDNA3.1(-) / Hygro* to generate *pcDNA3.1-Flag-HA / Hygro* plasmid. *HUWE1* ORF was then amplified by PCR and subcloned into *pcDNA3.1-Flag-HA / Hygro*, using primers *NotI*-hHuwe1-1 and *NotI*-hHuwe1-ORFT. For construction of *pEYFP-HUWE1*, *HUWE1* ORF was amplified by PCR using primers *BglII-hHUWE1-1* and *SacII-hHUWE1-2*, and subcloned into *pEYFP-C1* in between the *BglII* and *SacII* cloning sites. For construction of plasmid *pcDNA3.1-Flag-HA-CUL4B*, *CUL4B* ORF was amplified by PCR using primers *BamHI-hCUL4B* and *KpnI-hCUL4B*, and subcloned into *pcDNA3.1-Flag-HA / Hygro* in between the *BamHI* and *KpnI* cloning sites, using *Myc-pcDNA3-CUL4B* plasmid as template. *pcDNA3.1-Flag-HA-CUL4B / K859R* and *pcDNA3-Myc-CUL4B / K859R* plasmids were generated by site-specific mutagenesis PCR, using phusion High-Fidelity DNA polymerase (New England Biolabs) with primers *CUL4B/K859R-F* and *CUL4B/K859-R*.

Cell culture, Transfection and RNA Interference

HEK293T, MCF7, U2OS, HeLa and S3 HeLa cells were purchased from American Type Culture Collection. All cells were maintained in DMEM containing 10% fetal bovine serum (FBS) and penicillin-streptomycin at 37℃ in a 5% CO_2 incubator. Transient plasmid DNA transfection was performed with Lipofectamine 2000 (Invitrogen) according to the manufacturer's instructions.

For generating FH-CUL4B S3 HeLa stable cell line, S3 HeLa cells were transfected with *pcDNA3.1-Flag-HA-CUL4B* plasmid in 10 cm culture dishes. 24 h later, the cells were splitted in 60 mm plates and selected against hygromycin (700 μg/ml) for two weeks. Individual colonies were picked and expanded. Positive colonies were confirmed by western blotting and/or immunofluorescence with anti-HA monoclonal antibody.

Control, *CUL4B*, *ROC1*, *UBC12* and *DDB1* siRNA were synthesized from *Invitrogen Inc* or *Genepharma Inc*. The siRNA targeting sequences used are listed in table S2. Lipofectamine RNAi MAX (*Invitrogen*) was used for siRNA transfection. For transfection, cells were seeded into each well of a 6-well plate and subsequently transfected with a concentration of 100 nM siRNA using Lipofectamine RNAi MAX. 48 h post transfection, proteins were extracted, and the lysates were examined by western blotting.

Cycloheximide (CHX) Chase Assay

293T cells were transfected with *pcDNA3.1-Flag-HA-CUL4B* wild type or mutatant (K859R) constructs. 48 h after transfection, cells were treated with cycloheximide (100 μg/ml). HeLa cells were transfected with siRNA targeting *CUL4B*, *DDB1*, *ROC1* or *UBC12* and treated as above. Cells were harvested at the indicated times, and protein levels were evaluated by western blotting.

Immunoprecipitation and Western Blotting

Cells were harvested and lysed with NETENG-400 buffer (400 mM NaCl, 20 mM Tris-HCl, pH 7.4, 0.1% Nonidet P-40, 0.5 mM EDTA, 1.5 mM $MgCl_2$, 10% Glycerol), with freshly added phosphatase inhibitors and protease inhibitors cocktail (20 mM NaF, 1 mM Na_3VO_4, 1 mM PMSF, protease inhibitor cocktail (P8340, Sigma)). The lysates were diluted by adding 1.68 volume of the lysate of NETENG-0 (20 mM Tris-HCl, pH 7.4, 0.1% Nonidet P-40, 0.5 mM EDTA, 1.5 mM $MgCl_2$, 10% Glycerol) to obtain a final concentration of NaCl at 150 mM (NETENG-150: 150 mM NaCl, 20 mM Tris-HCl, pH 7.4, 0.1% Nonidet P-40, 0.5 mM EDTA, 1.5 mM $MgCl_2$, 10% Glycerol). The diluted lysates were mixed with 30 ul of Protein-G beads and CUL4B antibody (ab67035, Abcam) or HUWE1 antibody (ab70161, abcam) at 4 °C overnight. The proteins bound to the beads were washed three times with NETENG-150. The sample was eluted by 100 mM Glycine, pH 2.5, neutralized by adding 1M Tris-HCl, pH 8.5 (1/10 volume of elution buffer), and resolved by 4×Tris-acetate Sample Buffer (250 mM Tris-HCl, pH 8.5, 2% w/v Lithium Dodecyl Sulfate (LDS), 100 mM DTT, 0.4 mM EDTA, 10% (v/v) glycerol, 0.2 mM phenol red, 0.2 mM Brilliant Blue G-250) at 70 °C for 10 min, followed by 3-15% Tris-acetate SDS-PAGE, and analyzed by immunoblotting with the indicated antibodies. For all

those experiments except for immunoprecipitation, total cell lysates were obtained by using RIPA buffer (50 mM TrisCl (pH 8.0), 0.1% SDS, 150 mM NaCl, 1% Nonidet P-40, 0.5% sodium deoxycholate and protease inhibitor cocktail). For western blotting, the following antibodies were used: HUWE1 (ab70161, Abcam), CUL4B for immunoblotting (NBP1-40587, Novus Biologicals), CUL4B for immunoprecipatation (ab67035, Abcam), MCL-1 (559027, BD Pharmingen), c-Myc (ab32, Abcam), HA (MMS-101P, Convance), GFP (G6795, sigma), V5-Tag (042PM003, MBL), Normal rabbit IgG (sc-2027, Santa Cruz), Procaspase-3 (sc-7148, Santa Cruz), Active Caspase-3 (ab32042, Abcam).

Ubiquitination Assays

For *in vivo* ubiquitination assay, *pcDNA3-Myc-CUL4B* wild type, *K859R* mutant plasmids or *CUL4B* siRNA was co-transfected with *HA-Ubiquitin* and *His-V5 HUWE1* plasmids in 293T cells for 48 h. Cells were harvested and lysed in a phosphate/urea Buffer B (8 M Urea, 100 mM NaH_2PO_4， 10 mM Tris pH 8.0; 20 mM imidazole, 1 mM β-mercaptoethanol). The ubiquitinated proteins were precipitated with Ni-NTA agarose (QIAGEN), followed by four washes with Buffer C (8M Urea, 100 mM NaH_2PO_4，10 mM Tris-HCl，pH 6.3). The precipitated proteins were eluted with Buffer E (8 M Urea, 100 mM NaH_2PO_4, 10 mM Tris-HCl, pH 4.0; 250 mM imidazole; 1 mM β-mercaptoethanol), and boiled with 4×Tris-acetate sample buffer, resolved by 3-8% Tris-acetate SDS-PAGE, and analyzed by immunoblotting with the indicated antibodies.

For *in vitro* ubiquitination assay, GST-HUWE1 1-2500 (GST-HW2500) recombinant protein was produced from SF9 cells and CRL4B complex was purified from HEK293T cells. For GST-HW2500 expression and purification, Bac-to-Bac Baculovirus expression system (Invitrogen) was used according to the manufacturer’s instructions. Briefly, *pFastBac1-GST-HUWE1-1-2500* plasmid was constructed by introducing a GST-tag fused *HUWE1* DNA fragment encoding amino acids 1-2500 of HUWE1 protein into *pFastBac1*, and transformed into DH10Bac E.coli cells, selected with 50 ug/ml kanamycin, 7 ug/ml gentamicin, 10 ug/ml tetracycline, 100 ug/ml Bluo-gal, and 40 ug/ml IPTG. White colonies were confirmed by PCR and used for isolation of recombinant *GST-HW2500* Bacomid DNA. SF9 cells were transfected with *GST-HW2500* Bacomid DNA using Cellfectin (10362-100, Invitrogen) and baculovirus particles was generated and expanded. SF9 cells were infected with P2 stock

baculovirus *GST-HW2500* for 72 h before harvest and lysed with SF9 lysis buffer (50 mM Hepes pH 8.0, 120 mM NaCl, 0.5% NP-40, 1 mM PMSF, proteinase inhibitor cocktails) for 40 minutes. The whole lysates were centrifuged at 12,000 rpm for 30 minutes. The recombinant protein GST-HW2500 was pulled down with glutathione Sepharose 4B (Amersham bioscience) and eluted with GST elution buffer (100 mM Tris-HCl, pH 8.0, 15 mg glutathione/ml).

For purification of CRL4B ligase complex, 293T cells were transfected with *pcDNA3-Flag-HA-CUL4B*. 48 h after transfection, cells were lysed in NETENG-400 buffer (20 mM Tris-HCl, pH 7.4, 400 mM NaCl, 1 mM EDTA, 1.5 mM $MgCl_2$, 0.1% NP-40, 10% glycerol) for 30 minutes and clarified by centrifugation. The lysates were diluted by adding 1.68 lysate volume of NETENG-0 (20 mM Tris-HCl, pH 7.4, 0.1% Nonidet P-40, 0.5 mM EDTA, 1.5 mM $MgCl_2$, 10% Glycerol) to obtain a final concentration of NaCl at 150 mM (NETENG-150: 150 mM NaCl, 20 mM Tris-HCl, pH 7.4, 0.1% Nonidet P-40, 0.5 mM EDTA, 1.5 mM $MgCl_2$, 10% Glycerol). The diluted lysates were mixed with anti-FLAG M2 agarose for 4 h. Immobilized immunocomplex was eluted with elution buffer (50 mM TrisCl, pH 7.4, 100 mM NaCl, 10% Glycerol) containing FLAG peptide. Alternatively, Flag-HA-CUL4B complex can be eluted with 100 mM Glycine (pH 3.0) and neutralized by adding 1M Tris-HCl, pH 8.5 (1/10 volume of the elution buffer).

For *in vitro* ubiquitination assay, different combinations of E1 (100 ng, Boston Biochem), E2 (100 ng, Boston Biochem) and E3 (CRL4B) were mixed with 100 ng of recombinant GST-HW2500 substrate in a ubiquitin ligase reaction buffer (2 µg of HA-ubiquitin [Boston Biochem], 2 mM ATP, 5 mM $MgCl_2$, 2 mM DTT, 50 mM Tris-HCl, pH 7.4). Reactions were carried out for 90 minutes at 30ºC, terminated by boiling for 10 min in a SDS sample buffer, followed by 6% SDS-PAGE gel, and blotted with anti-HA and anti-HUWE1 antibodies.

DNA Damage Treatment

Cells were subjected to ionizing radiation using GSR-D1 137Cs gamma-irradiator (RPS Services Limited) at a dose rate of 1.8 Gy/min (8-Gy dose). For doxorubicin and etoposide treatments, cells were treated with either 0.5 ug /ml doxorubicin (Dox) or 10uM etoposide (Eto). The medium was changed after the treatment, and cells were incubated at 37°C to allow for DNA Repair.

Cell Viability and Apoptosis Assays

Cell viability was assessed indirectly by MTT assay. The cells were cultured in the 96-wells plates at a density of 5×10^4 ml^{-1} and treated with different concentration of doxorubicin, etoposide and cisplatin for 24 h. MTT was added to each well 4 h before termination of culture and incubated for 4 h at 37°C in 5% CO_2. 10% SDS was then added to each well, followed by overnight incubation at 37°C and 5% CO_2 to dissolve the dark blue crystal product. Each sample point was assayed with 4 replica points. Absorbance at 570 nm (A570) of the solubilized formazan was measured using a Bio-Tek Instruments (KC junior, USA) microplate reader to calculate inhibition rate for cell relative viability.

As for apoptosis assays, cells were suspended in PBS buffer and then washed, suspended in 100μl Annexin V-binding buffer. FITC-conjugated Annexin V and PI were used to stain the cells in each sample for 15 min at room temperature and analyzed by flow cytometry.

Gel analysis method with Image J

1. Open the file for analysis.
2. Choose the *Rectangular Selection tool*. Draw a rectangle around the first lane. Encompass some area of the lane above and below the band of interest.
3. Go to *Analyze>Gels>Select First Lane* (or press *Control + 1*).
4. Use the arrow keys to move the rectangle over the next lane and press *Control+2*.
5. When finished, press *Control + 3* (or go to *Analyze>Gels>Plot Lanes*).
6. Choose the *StraightLight selection tool*. At the base of each peak, draw a line from one side to the other to enclose the peak.
7. Choose the *MagicWand* and click inside the peak. A value will be caculated which represents the protein level of the band.

TABLES

Table S1.Primer Sequences of Construction of Plasmid Used in This Study

Primers	Sequence(5'to 3')	Notes
NotI-hHUWE1-1	ATAAGAATGCGGCCGCTGAAAGTAGACAGGACTAAACTG	Used for construction of plasmid
NotI-hHUWE1-ORFT	ATAAGAATGCGGCCGCTTAGGCCAGCCCAAAGCCTTC	*pcDNA31-Flag-HA-HUWE1*
BglII-hHUWE1-1	GAAGATCTAAAGTAGACAGGACTAAACTGAAG	Used for construction of plasmid *pEYFP-HUWE1*
SacII-hHUWE1-2	TCCCCGCGGTTAGGCCAGCCCAAAGCCTTC	
BamHI-hCUL4B	CGGGATCCGATGTCACAGTCATCTGGATCA	Used for construction of plasmid *pcDNA31-Flag-HA-Cul4B*
KpnI-hCUL4B	CGGGGTACCCTATGCAATATAGTTGTACTGGT	
BamHI-hCUL4B-F	CGGGATCCGATGATGTCACAGTCATCTGGATCAGG	Used for construction of plasmid *pCMV -Tag2A-Cul4B*
XhoI-hCUL4B-R	CCGCTCGAGCTATGCAATATAGTTGTACTGGTTT	
CUL4B-K859R-F	ATTGTTCGAATTATGAGAATGAGAAAGACACTT	Used for construction of *pcDNA3-Flag-HA-CUL4B/K859R*
CUL4B-K859R-R	AAGTGTCTTTCTCATTCTCATAATTCGAACAAT	or *pcDNA3-Myc-CUL4B/K859R* mutant plasmids

Table S2. Target Sequences of siRNA Used in This Study

siRNA	Targeted Sequence(5'to 3')	Notes
CUL4B-1	AAGCCUAAAUUACCAGAAA	
CUL4B-2	GGAGUUAUUUAGGGCUCAU	
CUL4B (3'UTR)	GGUUCUUACACACCAUUAATT	Target 3' untranslated region of Cul4B mRNA
DDB1	CCUGUUGAUUGCCAAAAAC	
ROC1	GAAGCGCUUUGAAGUGAAATT	
UBC12	GGGCTTCTACAAGAGTGGGAAGT	
HUWE1	AAUUGCUAUGUCUCUGGGACA	

FIGURE S1

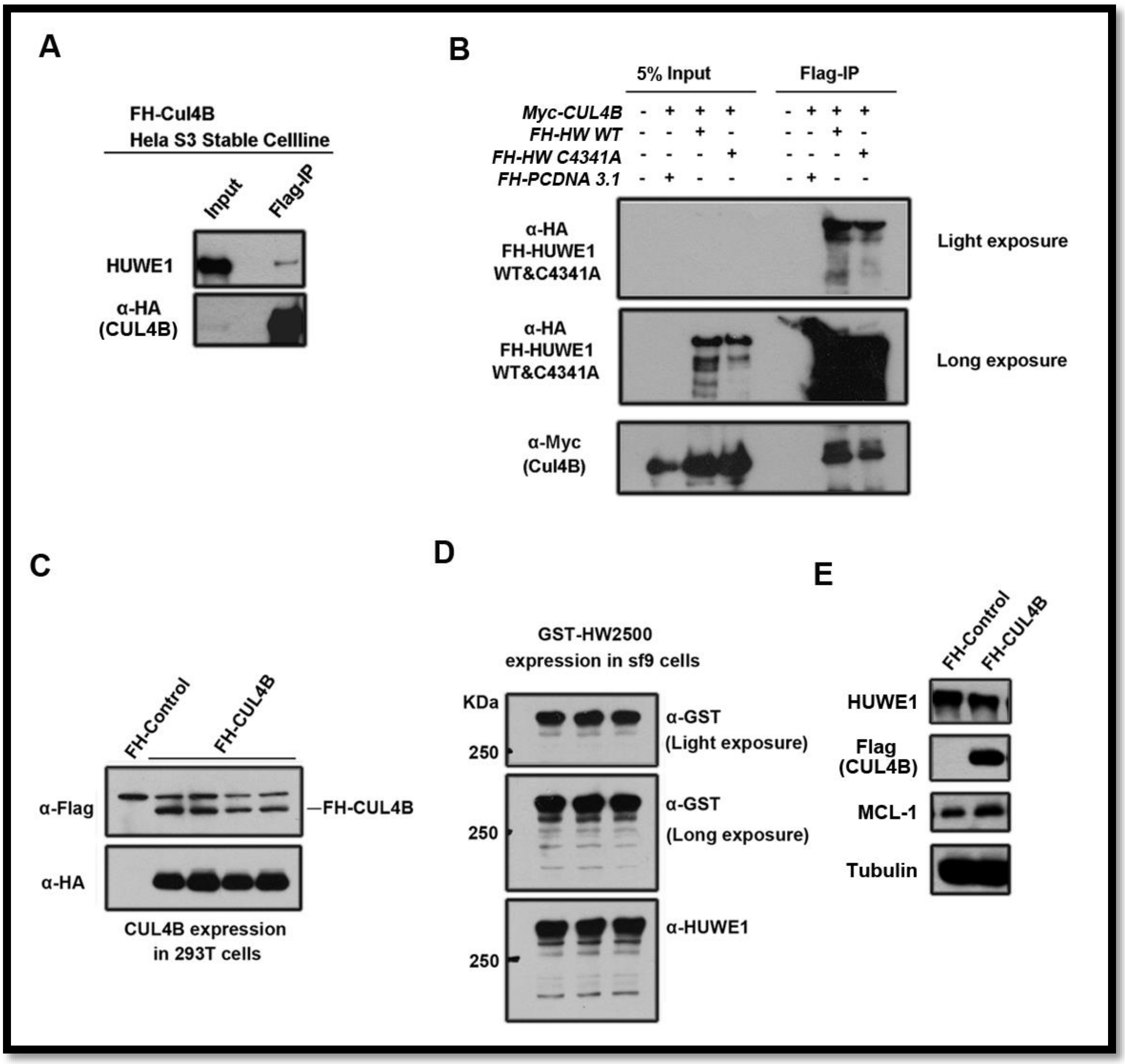

Fig S1: (A) The cell extracts of S3 HeLa cells with *pcDNA3.1-Flag-HA-CUL4B* stably transfected were immunoprecipitated with anti-FlAG-M2 agarose. The eluants were subjected to western blotting with anti-HA and anti-HUWE1 antibodies. (B) HeLa cells were co-transfected with *Myc-CUL4B* and *Flag-HA-HUWE1 WT*, or *Myc-CUL4B* and *Flag-HA-HUWE1 C4341A* mutant for 48 h. Either *Flag-HA-HUWE1 WT* or *Flag-HA-HUWE1 C4341A* mutant was immunoprecipitated with anti-FLAG M2 agarose, followed by western blotting with the indicated antibodies. (C) 293T cells were transfected with *pcDNA3.1-Flag-HA-CUL4B* plasmid for 48 h. Western blotting was utilized to evaluate the expression level of *Flag-HA-CUL4B* with anti-FLAG and anti-HA antibodies. (D) SF9 cells were

infected with P2 stock baculovirus *GST-HW2500* (*GST-HUWE1 1-2500*) for 72 h. The expression level of GST-HW2500 was evaluated through western blotting with the indicated antibodies. (E) 293T cells were transfected with *pcDNA3.1-Flag-HA* and *pcDNA3.1-Flag-HA-CUL4B*. The samples were harvested after 48 h transfection and analyzed by western blotting with the indicated antibodies.

FIGURE S2

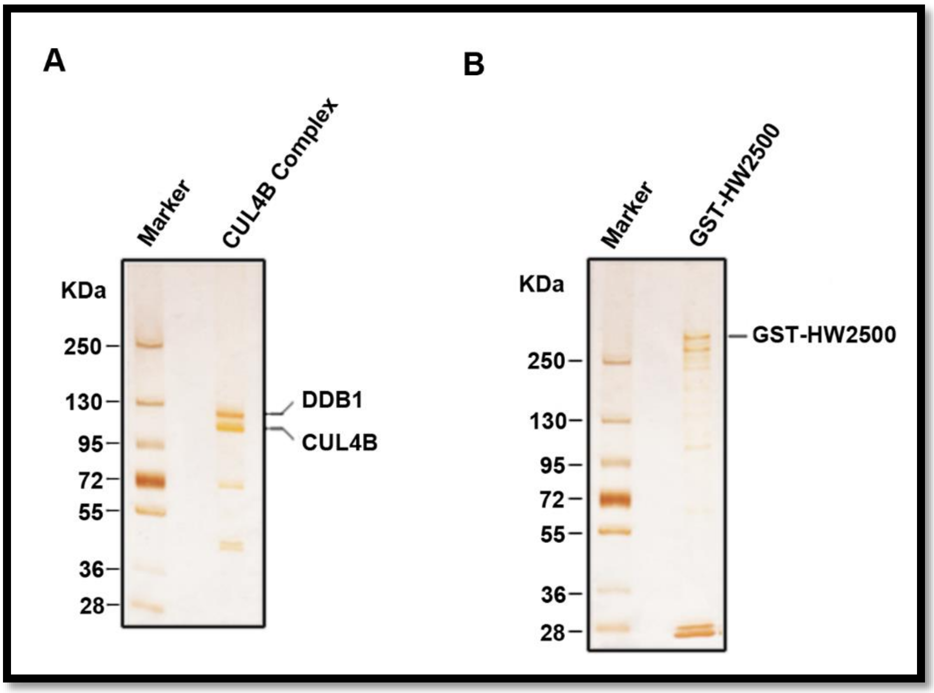

Fig S2: (A) Immunoaffinity purification of CUL4B-containing protein complexes. Cellular extracts from 293T cells transiently expressing FLAG-HA-CUL4B were immunopurified with anti-FLAG M2 Agarose gel and eluted with 100 mM Glycine, pH 3.0, and neutralized by Tris-HCl, pH 8.0. The eluates were resolved by 4%-12% SDS-PAGE, followed by silver-staining. (B) Silver staining of the purified, recombinant GST-HUWE1-1-2500 (GST-HW2500) protein. GST-HW2500 was expressed and purified from SF9 cells infected with baculovirus GST-HW2500 as descried in Experimental Procedures, and resolved by 4%-12% SDS-PAGE, followed by silver-staining.
